# Factors associated with common mental disorders: a study based on clusters of women

**DOI:** 10.11606/s1518-8787.2021055003124

**Published:** 2021-11-08

**Authors:** Cássio Zottis Grapiglia, Juvenal Soares Dias da Costa, Marcos Pascoal Pattussi, Vera Maria Vieira Paniz, Maria Teresa Anselmo Olinto

**Affiliations:** I Universidade do Vale do Rio dos Sinos Programa de Saúde Coletiva São Leopoldo RS Brasil Universidade do Vale do Rio dos Sinos. Programa de Saúde Coletiva. São Leopoldo, RS, Brasil; II Universidade do Vale do Rio dos Sinos Departamento de Saúde Coletiva São Leopoldo RS Brasil Universidade do Vale do Rio dos Sinos. Departamento de Saúde Coletiva. São Leopoldo, RS, Brasil

**Keywords:** Women, Mental Disorders, epidemiology, Risk Factors, Socioeconomic Factors, Latent Class Analysis

## Abstract

**OBJECTIVE:**

to identify factors associated with common mental disorders (CMD) in a sample of adult women in Southern Brazil.

**METHODS:**

This population-based study, composed of 1,128 women, investigated socioeconomic, behavioral and health/disease explanatory demographic variables. Five response groups were explored: one group with common mental disorders – cut-off point 6/7 in the *Self-Reporting Questionnaire* 20 (SRQ-20) – and four others corresponding to the different clusters found using the latent class clustering technique, also from the SRQ-20. These four clusters (low, medium-depressive, medium-digestive and high) were named (denominated) based on the mean scores in the SRQ-20 in each group and on the response patterns of the variables and factorial characteristics. The “low” cluster comprised women with lower SRQ-20 scores and, therefore less likely to present CMD. The “high” cluster, with high mean values in the SRQ-20, was related to higher psychiatric morbidity. We used the Poisson regression technique to compare the findings of the different groups.

**RESULTS:**

We identified ten variables as factors associated with CMD. Age, education, smoking, physical activity, perception of health and number of medical appointments were the common variables for the cut-off point and cluster-based analyses. Heavy alcohol use was associated only when the sample was evaluated as a cut-off point. Social class, work situation and existence of chronic diseases were associated only when the sample was analyzed by clusters. There was a significant association in the “high” cluster with lower classes (D or E), smoking, physical inactivity, existence of chronic diseases and negative perception of health.

**CONCLUSION:**

We identified different associated factors according to the response groups considered. New approaches allowing identification of subgroups of individuals with specific characteristics and associated factors may contribute for a more accurate understanding of CMD and provide the basis for health interventions.

## INTRODUCTION

The term “common mental disorders” (CMD) refers to a group of depressive symptoms, insomnia, fatigue, irritability, forgetfulness and difficulty to concentrate, as well as somatic complaints and the feeling of being useless^1^. Such disorders, more prevalent in women, cause suffering and functional disability and have an impact on several health, economic and social outcomes^2,3^.

We identified a prevalence rate of 17.6% in the year and 29.2% in life in a systematic review and meta-analysis study by Steel et al.^4^, who observed a consistent effect of the female sex in relation to mood disorders and anxiety. Studies conducted in Brazil have shown high prevalence in primary health care^5^ and higher odds ratio of CMD in women^6^.

The *Self-Reporting Questionnaire-20* (SRQ-20), one of the most recommended and used instrument for tracking common mental disorders, is validated also in Brazil^7^. Several studies of factors associated with CMD have used this instrument to define the presence of psychiatric morbidity based on the cut-off point criterion. In Brazil, the most used cut-off points for women have been 6/7^10^e 7/8^11^.

Following this criterion, studies have identified that female individuals^2,3,5,12^, non-white^2,13^, from socially underprivileged groups (lower social class and lower income, unemployed and with low education)^2,3,12,14^, smokers^13,15^, heavy alcohol users^16,17^, physically inactive^13^, with chronic diseases^13,18^ and with self-perception of bad health^13,19^ had higher prevalence of CMD. Moreover, greater recourse to health services, with more appointments per year, was also identified as a marker of CMD^20^. The relation between age^2,3,12,13,21^ and marital status^2,3,13,22^ with CMD has shown contradictory results.

We did not find studies exploring factors associated with CMD besides the traditional analysis of observational studies, which builds the outcome from cut-off points and may not recognize subgroups or clusters. The objective of this study is to investigate factors associated with CMD in a sample of adult women in Southern Brazil using the cut-off point and also identifying clusters or subgroups using the latent class analysis technique (with the SRQ-20 instrument), followed by a Poisson regression analysis.

## METHODS

The cross-sectional study “Condições de vida e saúde de mulheres adultas: estudo de base populacional no Vale dos Sinos – avaliação após 10 anos” – included a sample of 1,128 women in the town of São Leopoldo (RS). The research protocol was approved by the Unisinos Ethics Committee under No. 650,443. Each participant was informed about the objectives of the study and, after reading and signing a two-way informed consent form, underwent data collection.

The inclusion criteria were as follows: being a woman residing in one of the census tracts and households drawn from the São Leopoldo urban area, and being aged between 20 and 69 years. Women who did not reside in a drawn household at the time of the survey, women who were not physically and/or mentally fit to answer the questionnaire, or women who were pregnant were excluded.

Sampling was performed by conglomerates, with 40 census tracts drawn out of the 371 in the urban area of the town of São Leopoldo. The sample size of the baseline study was calculated to identify a risk ratio of 2.0, with a confidence level of 95% and statistical power of 80%, keeping an exposed: unexposed ratio of 2:1. We considered as unexposed those belonging in the higher education category (15 year of schooling of more). Based on these assumptions, we opted for the larger sample size calculated (1,013 women for the variable delayed cytopathological examination). We added 10% for possible losses/refusals and 15% to control confounding factors in the data analysis, totaling approximately 1,281 women. After considering the losses and refusals, which amounted to 11.9%, the final total number was 1,128 interviewed women.

The information was collected with questions of a designed instrument (covering demographic, socioeconomic and behavioral data on health/disease, use of health services, medicine, and health spending) and questions from validated instruments, of which we highlight the SRQ-20, applied to track CMD. The questionnaires were applied directly to the residents, in the households drawn. The fieldwork was conducted rigorously, and the interviewers had to undergo a training program with standardization of measurements and a pilot study. The database was double-typed for later comparison. We performed quality control using a summarized instrument composed of 10 questions applied to 10% of the sample, either by telephone or home visit.

Explanatory variables were as follows: age (20–29, 30–39, 40–49, 50–59, 60 years old or older), skin color (white and non-white), marital status (partner or no partner), education (4 years of schooling or less, 5 to 9 years of schooling, 10 to 12 years of schooling, 13 years of schooling or more), social class (A or B, C, D or E), work situation (working, retired/on medical leave/receiving a government allowance, housewife, unemployed), being the head of household or not, smoking (non-smoker, ex-smoker or smoker), heavy alcohol use, defined as ingesting more than 30 grams of alcohol a day (no or yes), recreational physical exercise, according to criteria of the International Physical Activity Questionnaire^23^(IPAQ) (physically active or inactive), previous pregnancies (none, one and two or more), existence of chronic diseases, referring to diabetes or high blood pressure (no disease, either disease or both diseases), health perception (positive or negative) and number of appointments per year (1, 2–5, 6–13, 14 or more appointments).

For this study, we built a summary database composed only of the exposure variables and the SRQ-20 instrument. Once the database was built, we identified clusters in the response patterns of the SRQ-20 instrument with a latent class analysis, using the Latent GOLD 5.1 software. This technique has advantages over conventional clustering techniques^24^. The criteria for setting the number of clusters were the Bayesian (BIC), the percentage of classification error and the residuals analysis^25^.

We opted for four clusters due to the lower BIC value and a satisfactory profile between the percentage of classification errors and the residuals profile. In addition to the criteria mentioned, we also considered the proportional number of women in each cluster. The clusters were named in reference to the mean SRQ-20 score and to the response patterns of the instrument variables.

Also in an exploratory manner, we performed a factor analysis of the instrument using a tetrachoric matrix and minimum values of 0.3 and 0.4 for factor loadings and communalities, respectively. The software used was FACTOR 10.9.02. This factor analysis identified two factors (Table 1). The first factor, called “depressive”, was composed of variables 6 (“Do you feel nervous, tense or worried?”), 9 (“Have you felt sad lately?”), 10 (“Have you cried more than usual?”), 11 (“Can you feel any pleasure in daily activities?”), 14 (“Do you feel useful in your life?”), 15 (“Have you been losing interest in life?”), 16 (“Do you feel like a worthless person?”), 17 (“Have you ever thought about ending your life?”) , 18 (“Do you feel tired all the time?”) , 20 (“Do you get tired easily?”). The second factor, called “digestive”, was composed of variables 7 (“Do you have poor digestion?”) and 19 (“Do you feel anything unpleasant in your stomach?”).

**Table 1:** Factor analysis using a tetrachoric matrix.

Variables	Factor 1 (Depressive)	Factor 2 (Digestive)	Commonality
6) Do you feel nervous, tense or worried?	0.468	0.267	0.416
7) Do you have poor digestion?	-0.108	0.888	0.702
9) Have you felt sad lately?	0.730	0.105	0.622
10) Have you cried more than usual?	0.665	0.018	0.455
11) Can you feel any pleasure in daily activities?	-0.848	0.157	0.610
14) Do you feel useful in your life?	-0.724	0.083	0.470
15) Have you lost interest in life?	0.936	-0.061	0.822
16) Do you feel that you are a worthless person?	0.854	-0.061	0.680
17) Have you ever thought about ending your life?	0.741	-0.106	0.481
18) Do you feel tired all the time?	0.674	0.191	0.620
19) Do you feel anything unpleasant in your stomach?	-0.070	0.971	0.879
20) Do you get tired easily?	0.578	0.284	0.580

Cluster 1, or “low”, had a mean SRQ of 1.94 (95% CI: 1.79–2.08), while the mean for cluster 4, or “high”, was 13.60 (95% CI: 13.25–13,93). The intermediate clusters (“medium-depressive” or 2, and “medium-digestive” or 3) had means close to the SRQ: 6.08 (95% CI: 5.87–6.29) and 7.63 (7.36–7.89), respectively, but with a more positive profile for the variables representative of the depressive factor in the first group and of digestive symptoms in the second group (Table 2). These variables called “depressive factor” and “digestive factor” were built out of the factor analysis of the instrument and had a maximum total value of 10 points and 2 points, respectively (Tables 1 and 2). After the clusters were identified, this database was migrated into SPSS and Stata 8.0 software, where we performed descriptive analyses of the sample as well as crude and adjusted analyses.

**Table 2:** Comparison between mean SRQ and digestive and depressive factors, according to the total sample and the different groups considered (n = 1,128).

	Sample Total (n = 1,128)	SRQ+ (Cut-off 6/7) (n = 450)	Cluster Low (n = 452)	Medium-Depressive Cluster (n = 240)	Middle-Digestive Cluster (n = 236)	Cluster High (n = 200)
Mean SRQ (95% CI)	6.07 (5.81–6.34)	10.76 (10.47–11.05)	1.94 (1.79–2.08)	6.08 (5.87–6.29)	7.63 (7.36–7.89)	13.60 (13.25–13.93)
Digestive factor^a^ (95% CI)	0.64 (0.60–0.69)	1.13 (1.05–1.21)	0.22 (0.17–0.27)	0.03 (0.01–0.04)	1.64 (1.57–1.70)	1.18 (1.06–1.30)
Depressive factor^b^ (95% CI)	2.86 (2.71–3.01)	5.28 (5.08–5.49)	0.69 (0.62–0.76)	3,30 (3,14–3,46)	2,82 (2,66–2,98)	7,30 (7,10–7,49)

^a^ Digestive factor: variable with values between 0-2, according to factorial findings in the sample.

^b^ Depressive factor: variable with values between 0-10, according to factorial findings in the sample.

Regarding the response variables (response groups), 5 groups were considered. The first was the positive for CMD group, considering the cut-off point 6/7^10^. We used this group as a reference and more classic way of investigating associated factors in relation to the subject. The other four response groups were the four clusters described. We evaluated the association between explanatory variables and response variables (response groups) by prevalence ratios (PR) and confidence intervals (95% CI). The crude and adjusted analyses were performed by Poisson regression, with control for the design effect. In the adjusted analyses, all variables were initially included, sequentially removing from the analyses those that did not present a minimum p-value of 0.05. Finally, only significant variables remained in each of the five response groups considered.

## RESULTS

When considering the different clusters, 40.1% of the women belonged in the “low” cluster, 21.3% in the “medium-depressive” cluster, 20.9% in the “medium-digestive” cluster, and 17.7% in the “high” cluster. As Table 3 shows, 39.9% of the women presented a positive CMD criterion, according to the cut-off point 6/7 in the SRQ-20 instrument (Table 3).

**Table 3:** Descriptive analysis of socioeconomic, demographic, behavioral, health and disease variables in the sample of women from the town of São Leopoldo, Southern Brazil (n = 1,128).

Groups	n	% SRQ+ (Cut-off 6/7)	% Low Cluster	% Medium-Depressive Cluster	% Medium-Digestive Cluster	% High Cluster
Variables	1,128	39.9	40.1	21.3	20.9	17.7
Age (years)						
20–29	216	18.2	20.1	**18.3**	19.9	17.0
30–39	244	24.2	16.8	**30.8**	24.6	18.0
40–49	276	23.8	24.3	**24.6**	22.9	26.5
50–59	228	19.3	23.0	**14.2**	18.2	23.5
60 or older	164	14.4	15.7	**12.1**	14.4	15.0
Skin color						
White	840	72.7	76.3	72.9	74.2	72.5
Non-white	288	27.3	23.7	27.1	25.8	27.5
Marital status						
Partner	720	62.9	62.4	67.9	65.3	60.5
No partner	408	37.1	37.6	32.1	34.7	39.5
Education (years)						
13 or more	114	**4.8**	**14.6**	8.0	11.0	**2.1**
10–12	391	**30.6**	**38.4**	35.9	35.2	**27.1**
5–9	417	**40.4**	**34.8**	40.5	32.6	**46.4**
4 or less	188	**24.3**	**12.1**	15.6	21.2	**24.5**
Social class						
A or B	390	**25.7**	**40.6**	35.7	35.2	**20.0**
C	596	**58.0**	**49.8**	53.4	54.2	**59.0**
D or E	136	**16.3**	**9.6**	10.9	10.6	**21.0**
Work situation						
Working	637	**50.3**	**61.9**	56.5	58.5	**42.5**
Retired/On leave.^a^	186	**16.7**	**17.7**	13.8	14.4	**19.5**
Housewife	182	**18.7**	**13.5**	17.6	14.0	**23.0**
Unemployed	121	**14.3**	**6.9**	12.1	13.1	**15.0**
Head of household						
No	544	**44.0**	50.9	50.0	47.5	**41.0**
Yes	584	**56.0**	49.1	50.0	52.5	**59.0**
Smoking						
No	661	**54.0**	**62.4**	61.3	58.5	**47.0**
Ex-smoker	259	**21.8**	**23.9**	21.3	26.3	**19.0**
Yes	208	**24.2**	**13.7**	17.5	15.3	**34.0**
Heavy alcohol use						
No	1087	95.8	97.3	95.8	98.3	96.0
Yes	34	4.2	2.7	4.2	1.7	4.0
Physical activity						
Yes	162	**8.4**	**20.8**	12.1	12.7	**4.5**
No	966	**91.6**	**79.2**	87.9	87.3	**95.5**
Pregnancies						
None	172	**11.3**	**18.8**	15.4	15.3	**7.0**
1	254	**19.8**	**23.7**	25.4	22.0	**17.0**
2 or more	702	**68.9**	**57.5**	59.2	62.7	**76.0**
Chronic diseases						
None	785	**62.7**	**75.5**	75.3	69.8	**51.8**
One	269	**28.8**	**20.5**	18.4	24.7	**38.0**
Two	66	**8.5**	**4.0**	6.3	5.5	**10.2**
Perception of health						
Positive	748	**45.6**	**83.4**	66.3	62.7	**32.0**
Negative	380	**54.4**	**16.6**	33.8	37.3	**68.0**
Medical appointments / year						
None	174	**16.0**	**14.6**	18.8	10.2	**19.6**
1	179	**11.4**	**20.6**	15.0	11.9	**11.1**
2–5	277	**19.6**	**27.9**	23.3	27.5	**15.0**
6–13	172	**17.1**	**14.4**	14.6	15.7	**17.6**
14 or more	325	**35.9**	**22.6**	28.3	34.7	**36.7**

^a^ Retired / On leave: this category included women who are retired, on medical or maternity or receiving a government allowance. The numbers in bold refer to significant variables (p < 0.05) in each group considered.

Regarding socioeconomic and demographic variables, intermediate ages, between 30 and 49 years, were prevalent when the cut-off group was considered. Regarding the clusters, higher ages tended to be more prevalent in women with a higher SRQ-20. White skin color predominated in all groups, as did the marital status of having a partner. When analyzing education, the most educated group (13 years or more) was the least prevalent in the group with SRQ+ in relation to the cut-off point. In the clusters, education varied inversely with the highest SRQ-20 score. The least privileged social classes (D or E) were more prevalent in the “high” cluster. Class C was the most prevalent in all clusters and also when considering the cut-off point. Regarding work situation, the “working” status prevailed in all groups, and the distribution of women with the “head of household” status was similar, with a slight increase in prevalence in the cut-off point groups and in the “high” cluster (Table 3).

When considering the behavioral variables, non-smoking and physically inactive women predominated in all groups. Heavy alcohol use (> 30g of alcohol per day) had very little prevalence in all groups (Table 3).

Most women reported not having any chronic disease (diabetes or high blood pressure). Most also reported two or more pregnancies. Perception of positive health varied inversely with the clusters regarding the highest score in SRQ-20: the highest frequency (83.4%) was found in the “low” cluster, and the lowest (32%) in the “high” cluster. The indicator of the number of medical appointments per year varied according to the group, and it was distributed more irregularly (Table 3).

In the crude and adjusted analyses, the cluster with the highest protection for CMD, which was called “low”, presented a relationship between age, education, work situation, physical activity, perception of health and number of medical appointments per year (Table 4). Social class, smoking, physical activity, existence of chronic diseases and perception of health were associated with the “high” cluster (Table 4). In the intermediate clusters, only two factors were identified: age in the “medium-depressive” cluster, and number of medical appointments per year in the “medium-digestive” cluster (Table 5).

**Table 4:** Crude and adjusted analysis of groups with SRQ + according to cut-off point 6/7, “low” cluster and “high” cluster.

Groups	SRQ + (Cut-off 6/7)		Cluster 1 (Low)		Cluster 4 (High)	
		
	PR Crude	Adjusted PR	PR Crude	Adjusted PR	Crude PR	Adjusted PR
Variables						
Age (years)						
20–29	1	1	1	1		
30–39	1.18 (0.92–1.51)	1.20 (0.94–1.53)	0.74 (0.57–0.96)	0.73 (0.56–0.95)		
40–49	1.02 (0.79–1.32)	0.90 (0.71–1.16)	0.95 (0.76–1.17)	0.98 (0.79–1.21)		
50–59	1.01 (0.74–1.37)	0.79 (0.60–1.02)	1.08 (0.85–1.37)	1.24 (1.00–1.53)		
60 or older	1.04 (0.79–1.39)	0.73 (0.54–0.99)	1.03 (0.79–1.33)	1.23 (0.93–1.61)		
Education (years)						
13 or more	1	1	1	1		
10–12	1.87 (1.23–2.86)	1.59 (1.07–2.37)	0.77 (0.64–0.92)	0.90 (0.75–1.08)		
5–9	2.32 (1.48–3.62)	1.71 (1.12–2.60)	0.65 (0.54–0.78)	0.84 (0.70–1.00)		
4 or less	3.09 (1.92–4.98)	2.20 (1.44–3.35)	0.50 (0.35–0.73)	0.68 (0.48–0.95)		
Social class						
A or B						
C					1.93 (1.31–2.85)	1.16 (0.80–1.69)
D or E					3.01 (2.05–4.43)	1.76 (1.19–2.58)
Work situation						
Working			1	1		
Retired/On leave^a^			0.98 (0.84–1.15)	1.06 (0.91–1.24)		
Housewife			0.77 (0.59–1.00)	0.88 (0.67–1.15)		
Unemployed			0.58 (0.43–0.80)	0.70 (0.53–0.92)		
Smoking						
No	1	1			1	1
Ex-smoker	1.03 (0.87–1.22)	0.97 (0.82–1.14)			1.03 (0.77–1.38)	0.92 (0.69–1.22)
Yes	1.43 (1.24–1.65)	1.13 (0.99–1.31)			2.30 (1.79–2.95)	1.67 (1.30–2.16)
Heavy alcohol use						
No	1	1				
Yes	1.42 (1.05–1.90)	1.41 (1.11–1.79)				
Physical activity						
Ativo	1	1	1	1	1	1
Inactive	1.82 (1.33–2.49)	1.35 (1.00–1.82)	0.64 (0.54–0.76)	0.78 (0.67–0.92)	3.56 (1.87–6.77)	2.19 (1.19–4.02)
Chronic diseases						
None					1	1
One					2.15 (1.61–2.87)	1.49 (1.12–1.98)
Two					2.33 (1.44–3.78)	1.36 (0.84–2.21)
Perception of health						
Positive	1	1	1	1	1	1
Negative	2.35 (2.06–2.68)	2.09 (1.86–2.34)	0.39 (0.32–0.49)	0.44 (0.35–0.55)	4.18 (3.15–5.55)	3.14 (2.31–4.27)
Medical appointments / year						
None	1	1	1	1		
1	0.69 (0.52–0.91)	0.74 (0.55–1.01)	1.37 (1.08–1.74)	1.29 (1.02–1.64)		
2–5	0.77 (0.61–0.96)	0.87 (0.71–1.07)	1.20 (0.96–1.50)	1.08 (0.86–1.36)		
6–13	1.08 (0.87–1.34)	1.21 (0.98–1.50)	1.00 (0.75–1.33)	0.93 (0.70–1.22)		
14 or more	1.20 (0.96–1.50)	1.19 (0.95–1.50)	0.83 (0.63–1.09)	0.83 (0.63–1.09)		

PR: prevalence ratio.

^a^ Retired / On leave: this category included women who are retired, on medical or maternity leave or receiving a government allowance.

**Table 5:** Associated factors in relation to intermediate clusters.

Groups	Cluster 2 (Medium-Depressive)		Cluster 3 (Medium-Digestive)	
	
	Crude PR	Adjusted PR	Crude PR	Adjusted PR
Variables				
Age^a^ (years)				
20–29	1	1		
30–39	1.49 (1.06–2.09)	1.49 (1.06–2.09)		
40–49	1.05 (0.73–1.51)	1.05 (0.73–1.51)		
50–59	0.73 (0.48–1.12)	0.73 (0.48–1.12)		
60 or older	0.87 (0.57–1.33)	0.87 (0.57–1.33)		
Appointments/year^b^				
None			1	1
1			1.13 (0.69–1.85)	1.13 (0.69–1.85)
2–5			1.70 (1.08–2.67)	1.70 (1.08–2.67)
6–13			1.56 (0.96–2.52)	1.56 (0.96–2.52)
14 or more			1.83 (1.12–2.98)	1.83 (1.12–2.98)

PR: prevalence ratio.

^a^ The only significant variable in cluster 2.

^b^ The only significant variable in cluster 3.

When considering the cut-off point group, we found a relationship between age, education, smoking, heavy alcohol use, physical activity, perception of health and number of medical appointments per year. In this group, the lowest prevalence ratios were found in older women. In the age category between 30 and 39 years, we found prevalence 20% higher than the reference category (being aged between 20 and 29 years). The “low” cluster showed a consistent trend of increasing prevalence of women as age increased. The prevalence ratio was 1.24 in the group of women aged between 50 and 59 years (Table 4).

Schooling proved to be a relevant associated factor when considering the cut-off point, with 2.2 times higher prevalence in the group between 0 and 4 years of schooling compared to the reference category of 13 years of schooling or more. This variable was also significant in the “low” cluster, as we found the lowest prevalence ratio (0.68) among women with the lowest schooling. This demonstrates the protective effect of the variable in relation to CMD. Social class showed an association only in the “high” cluster, with a prevalence ratio of 1.76 in the lower classes (D or E). Work situation, one more related socioeconomic variable, showed an inverse linear trend toward work-unemployment in the “low” cluster. The prevalence ratio was a lower (0.70) and the significance was higher among unemployed women (Table 4).

In the behavioral variable that considered smoking, the prevalence ratios were significant in the category of smokers, both in the cut-off point group (PR = 1.13) and in the “high” cluster (PR = 1.67). Heavy alcohol use (intake > 30 grams of alcohol/day) was only associated with the cut-off point group. The prevalence ratio was 1.41.

Regarding physical activity, there was an association between being physically inactive in the cut-off point groups (PR = 1.35), “low” cluster (PR = 0.78) and “high” cluster (PR = 2.19), with all confidence intervals consistent (Table 4). Similarly and significantly, the perception of health variable showed associations in the three groups. The lowest prevalence ratio of a negative perception was found in the “low” cluster (PR = 0.44), and the highest in the “high” cluster (PR = 3.14), The intermediate value was found in the cut-off point group (PR = 2.09) (Table 4).

Existence of chronic diseases was significant only in the “high” cluster, with slightly higher prevalence in the category “having one chronic disease” (PR = 1.49) compared to “having two” (PR = 1.36) (Table 4).

With regard to medical appointments, the cut-off point group showed a lower prevalence trend in relation to the reference (no appointments) in the categories between 1 and 5 appointments, and increasing prevalence in relation to a greater number of appointments (6 or more). The “low” cluster showed significance in the categories of one appointment, and 14 or more appointments. The prevalence ratios were 1.29 and 0.83, respectively (Table 4). The “medium-digestive” cluster showed a trend of progressive prevalence ratios in the same direction as the categories with the highest number of appointments. The highest value found (PR = 1.83) was the one corresponding to the category with the most appointments: 14 or more (Table 5).

We also investigated the mean SRQ-20 in each analyzed group, as well as digestive (variables 7 and 19) and depressive (variables 6, 9, 10, 11, 14, 15, 16, 17, 18 e 20) factors, defined according to factor analysis of the sample (Tables 1 and 2). Regarding the digestive factor, we observed that the “medium-digestive” cluster was the one with the highest score in this factor, higher even than the “high” cluster. The “medium-depressive” cluster was the one with the lowest score in this factor, lower even than the “low” cluster (Table 2).

## DISCUSSION

The literature indicates a significant association between CMD and the female sex. Mood disorders and anxiety are more prevalent in women compared to men, who in turn are more likely to present disorders related to substance use^4^. Studies that aim to identify CMD associated factors with mental health screening instruments and their response patterns, based on specific population bases of women, add data to this context. When considering the classic view of the analysis of the factors associated with CMD based on a cut-off point, we should bear in mind that within the groups without the disease (SRQ-) or with the disease (SRQ+) – represented in this study by psychiatric morbidity –, there may be different subgroups or clusters with particular characteristics and specific associated factors.

The use of the traditional cut-off point can cause associated factors to be assigned or identified in groups that, however heterogeneous, will be “homogenized” by this methodology. The evaluation by subgroups or clusters based on the response patterns of SRQ-20 allowed us to visualize in which group each associated factor exhibited relevance. This new insight may, therefore, result in more specific studies on CMD and associated factors, questioning the traditional cut-off point.

When considering age as a factor associated with CMD, the literature has shown contradictory results^2,3,12,13,21^. Most studies reviewed found a consistent increase in the prevalence of CMD with age, which is not a finding of this study. The results found here likely reflect the increasing demands and increasing stress among younger women^13^. Due to the analysis used, which included clusters, we observed a protective effect of age in relation to CMD, with a tendency to increase the presence of older women in the SRQ-20 lowest-scoring group (“low” cluster). However, in clusters with greater probability of presenting CMD, the trend proved to be inverse. Thus, the prevalence of older women decreased as the scores in the SRQ-20 progressed (“medium-depressive” cluster and cut-off point group).

Studies have shown that social disadvantages such as low education, income, class, and unemployment remain the most consistent risk factors for CMD^2,3,12,14^. Although social class and education are related variables, in this study, these two variables presented different influence when the clusters were evaluated. As a result, higher education had a protective effect for CMD, as shown in the “low” cluster, as well as the context of labor history. The literature describes that working is supposed to have a protective effect against CMD on women, as opposed to being a housewife or being unemployed^3,21^. Social class was the only socioeconomic variable identified in the group with the highest probability of psychiatric morbidity (“high” cluster), which shows a trend of higher prevalence among the lower social classes, with a significant prevalence ratio of 1.76 in classes D or E.

In the cut-off point group, only schooling was important as a socioeconomic variable, with evidence of consistently increasing prevalence as schooling decreases. Consequently, women with worse economic or socially underprivileged conditions should experience greater mental suffering. With these data, we can assume that lower unemployment rates, increased education and access to more privileged classes would improve mental health. Each of these variables has a different influence on each group considered.

Regarding smoking, the analysis showed significance in the category of women who are currently smokers in the cut-off point group, corroborating other studies conducted in Southern Brazil^13,20^. This association was also found in the “high” cluster, but with greater magnitude. Thus, we infer that the influence of smoking occurred mainly among women with the highest SRQ, and not in intermediate values, as the cut-off point methodology might suggest.

Studies have also identified alcoholism and its relationship with CMD^13,20,26^. With the cut-off point criterion, CMD was associated with the behavioral variable of heavy alcohol use (> 30g alcohol/day). This relationship was not shown in the analysis according to the clusters, which demonstrates that the analysis based on the traditional cut-off point found different results on account of submitting women with different values of SRQ+ (which could vary from 7 to 20) to the same analysis.

The physical activity and health perception variables were significant both in the cut-off group and in the end clusters (“low” and “high”). As the literature describes, we found a beneficial effect of physical activity in relation to physical and psychosocial well-being, which is reflected in the prevalence ratios in the “low” cluster. Muscle growth and the decreased percentage of fat, optimization of cardiorespiratory conditions, decreased anxiety and depression – which impact on mood and self-esteem – are some of the possible protective effects against CMD and several chronic diseases^13^. Corroborating other studies, we found a higher prevalence of physically inactive women in the cut-off point groups and the “high” cluster, which presented higher psychiatric morbidity^13,26^. Self-perception of health considered negative presented higher prevalence ratios in the SRQ-20 highest scoring groups (cut-off point and “high” cluster), agreeing with findings in the literature^2,13,21^.

Regarding the existence of chronic diseases, international research by the World Health Organization (WHO) in 17 countries showed an association between CMD and several chronic pathologies, such as diabetes, asthma, hypertension, arthritis, ulcer and heart disease^27^. Though the literature indicates an association between CMD and chronic conditions with the traditional cut-off point, this study only found an association with these conditions in the higher SRQ group (“high”). This finding suggests that measures that optimize chronic disease management may have an impact among women with higher psychiatric morbidity. In the “high” cluster, the concomitance of behavioral factors (smoking and physical inactivity) with health and disease variables (negative self-perception of health and chronic diseases) was clearly characterized.

The highest scoring cluster in the digestive factor (“medium-digestive”) presented a progressive relationship with the number of medical appointments. According to Bekhuis et al.^28^, there are different combinations between depressive, anxious and somatic symptoms, which impacts on the demand for health services. In this sample, digestive symptoms motivated a greater number of medical appointments than the SRQ-20 score itself or depressive symptoms. Thus, these digestive symptoms require special care and attention to complex interpretations arising from the expressed symptoms, in order to better understand this demand.

In summary, considering the associated factors found, the “low” cluster is comprised mostly of women who are older, more educated, physically active, and have a work history, positive self-perception of health and a tendency to have few medical appointments. The “high” cluster consists of women who are from the lower classes, smokers, physically inactive and have chronic diseases and a negative self-perception of health. Women with a tendency to seek more for medical appointments, intermediate SRQ-20 values and greater expression of digestive symptoms in general belong in the middle-digestive cluster. The middle-depressive cluster, also with intermediate SRQ-20 values, is comprised of young women who report symptoms more consistent with the depressive sphere. The cut-off point group is comprised of younger women who are heavy alcoholics and smokers, physically inactive, have low education, a self-perception of poor health and a greater tendency to require medical appointments.

As limitations of this study, we can mention the impossibility of extending its findings to the general population, as the sample was restricted to women. There is also the possibility of reverse causality, since this is a cross-sectional study. In spite of this, studies of this kind are important tools to identify and describe risk groups, thus contributing to the planning of health care actions^29,30^.

## CONCLUSION

This study, which used latent class cluster analysis, presents a fresh, one-of-a-kind perspective on CMD and associated factors. The characterization of subgroups, defined by the response profile of the SRQ-20 instrument, allowed finding particular associated factors, which provides more specific results. In this perspective, and considering the relevance of the subject, further studies using this methodology are necessary.
