# Supplementary material for: Factors associated with common mental disorders: a study based on clusters of women
Source: Rev Saude Publica. 2021 Nov 8;55:77. doi: 10.11606/s1518-8787.2021055003124 (PMC8577533; doi:10.11606/s1518-8787.2021055003124)
Supplement: Translation

# Translation: Fatores associados aos transtornos mentais comuns: estudo baseado em clusters de mulheres

**Authors:** Cássio Zottis Grapiglia, Juvenal Soares Dias da Costa, Marcos Pascoal Pattussi, Vera Maria Vieira Paniz, Maria Teresa Anselmo Olinto

**Affiliations:** I Brasil Universidade do Vale do Rio dos Sinos. Programa de Saúde Coletiva. São Leopoldo, RS, Brasil; II Brasil Universidade do Vale do Rio dos Sinos. Departamento de Saúde Coletiva. São Leopoldo, RS, Brasil

**Keywords:** Mulheres, Transtornos Mentais, epidemiologia, Fatores de Risco, Fatores Socioeconômicos, Análise de Classes Latentes

## Abstract

**OBJETIVO:**

Identificar fatores associados aos transtornos mentais comuns (TMC) em uma amostra de mulheres adultas no Sul do Brasil.

**MÉTODOS:**

Em estudo de base populacional composto por 1.128 mulheres, foram investigadas variáveis explicativas demográficas, socioeconômicas, comportamentais e de saúde/doença. Cinco grupos-resposta foram explorados: um grupo com transtornos mentais comuns – ponto de corte 6/7 no *Self-Reporting Questionnaire* 20 (SRQ-20) – e outros quatro correspondentes aos diferentes *clusters* encontrados através da técnica de clusterização por classe latente, também a partir do SRQ-20. Esses quatro *clusters* (baixo, médio-depressivo, médio-digestivo e alto) foram denominados com base nas pontuações médias do SRQ-20 em cada grupo e nos padrões de resposta das variáveis e características fatoriais. O *cluster* “baixo” compreendeu mulheres com menores escores no SRQ-20 e, portanto, com menor probabilidade de apresentarem TMC. Já o *cluster* “alto”, com valores médios elevados no SRQ-20, esteve relacionado a maior morbidade psiquiátrica. Utilizou-se a técnica de regressão de Poisson, com comparação entre os achados dos diferentes grupos.

**RESULTADOS:**

Foram identificadas 10 variáveis como fatores associados aos TMC. Idade, escolaridade, tabagismo, atividade física, percepção de saúde e número de consultas foram as variáveis comuns para as análises com ponto de corte assim como nas baseadas em *clusters*. Etilismo pesado esteve associado somente quando avaliada a amostra como ponto de corte. Já classe econômica, situação laboral e presença de doenças crônicas estiveram associadas somente quando analisada a amostra por *clusters*. No *cluster* “alto”, houve associação significativa com classes econômicas mais baixas (D ou E), tabagismo, inatividade física, presença de doenças crônicas e percepção de saúde negativa.

**CONCLUSÕES:**

Diferentes fatores associados foram identificados segundo os grupos-resposta considerados. Novos enfoques que possibilitem identificar subgrupos de indivíduos com características e fatores associados específicos poderiam contribuir para um entendimento mais apurado dos TMC, servindo de base para intervenções em saúde.

## INTRODUÇÃO

O termo “transtornos mentais comuns” (TMC) refere-se a um conjunto de sintomas depressivos, insônia, fadiga, irritabilidade, esquecimento e dificuldade de concentração, além de queixas somáticas e sentimento de inutilidade^1^. Esses transtornos, mais prevalentes em mulheres, causam sofrimento e incapacidade funcional, além de impactarem em diversos desfechos de saúde, econômicos e sociais^2,3^.

Foram identificadas prevalências de 17,6% no ano e 29,2% na vida em estudo de revisão sistemática e metanálise de Steel et al.^4^ que observou efeito consistente do sexo feminino em relação a transtornos de humor e ansiedade. Estudos realizados no Brasil demonstraram prevalência elevada na atenção primária em saúde^5^ e maior razão de chance de TMC em mulheres^6^.

O *Self-Reporting Questionnaire-20* (SRQ-20), um dos instrumentos mais recomendados e utilizados para rastrear transtornos mentais comuns, encontra-se validado inclusive no Brasil^7^. Diversos estudos de fatores associados aos TMC utilizaram esse instrumento para definir a presença de morbidade psiquiátrica, baseando-se no critério do ponto de corte. No Brasil, os pontos de corte mais utilizados para as mulheres têm sido 6/7^10^e 7/8^11^.

Seguindo esse critério, estudos identificaram que indivíduos de sexo feminino^2,3,5,12^, de cor não branca^2,13^, pertencentes a grupos com desvantagem social (classe social mais baixa e menor renda, desempregados e com escolaridade baixa)^2,3,12,14^, tabagistas^13,15^, etilistas pesados^16,17^, inativos fisicamente^13^, com doenças crônicas^13,18^ e com autopercepção de saúde ruim^13,19^ apresentaram maiores prevalências de TMC. Além disso, a maior utilização dos serviços de saúde, com mais consultas/ano, também foi identificada como marcador de TMC^20^. A relação da idade^2,3,12,13,21^ e do estado conjugal^2,3,13,22^ com os TMC tem demonstrado resultados contraditórios.

Não foram encontrados estudos explorando fatores associados aos TMC além da tradicional análise de estudos observacionais, que constrói o desfecho a partir de pontos de corte e pode não reconhecer subgrupos ou *clusters*. O presente estudo propõe-se a investigar fatores associados aos TMC em uma amostra de mulheres adultas no Sul do Brasil, utilizando o ponto de corte e também identificando *clusters* ou subgrupos pela técnica de análise de classe latente (com o instrumento SRQ-20), seguida de análise de regressão de Poisson.

## MÉTODOS

O estudo transversal “Condições de vida e saúde de mulheres adultas: estudo de base populacional no Vale dos Sinos – avaliação após 10 anos” contemplou amostra de 1.128 mulheres no município de São Leopoldo (RS). A pesquisa teve protocolo aprovado pelo Comitê de Ética da Unisinos sob o nº 650.443. Cada participante foi esclarecida a respeito dos objetivos do estudo e, após leitura e assinatura de termo de consentimento livre e esclarecido em duas vias, procedeu-se a coleta de dados.

Estabeleceram-se como critérios de inclusão: ser mulher residente em algum dos setores censitários e domicílios sorteados da zona urbana de São Leopoldo e ter idade entre 20 e 69 anos. As mulheres que, apesar de estarem no domicílio sorteado à época da pesquisa, não residiam nesse domicílio, assim como aquelas que não apresentaram condições físicas/mentais para responder o questionário, ou que estavam grávidas, foram excluídas.

Foi realizada amostragem por conglomerados, com 40 setores censitários sorteados entre os 371 da zona urbana do município de São Leopoldo. O tamanho da amostra do estudo de base foi calculado para identificar uma razão de risco de 2,0, com um nível de confiança de 95% e poder estatístico de 80%, mantendo razão de expostos: não expostos de 2:1. Consideraram-se não expostos os pertencentes à categoria de maior escolaridade (15 ou mais anos de estudo). Com base nesses pressupostos, optou-se pelo maior tamanho de amostra calculado (1.013 mulheres para a variável exame citopatológico atrasado). Foram acrescentados 10% para eventuais perdas/recusas e 15% para controlar fatores de confusão na análise dos dados, totalizando aproximadamente 1.281 mulheres. Após consideradas as perdas e recusas, que corresponderam a 11,9%, o número total final foi de 1.128 mulheres entrevistadas.

As informações foram coletadas por meio de questões de instrumento construído (abarcando dados demográficos, socioeconômicos e comportamentais sobre saúde/doença, utilização de serviços sanitários, medicamentos e gastos em saúde) e de questões pertencentes a instrumentos validados, destacando-se o SRQ-20, aplicado para rastrear TMC. Os questionários foram aplicados diretamente às residentes, nos domicílios sorteados. Conduziu-se o trabalho de campo com rigor, e os entrevistadores foram submetidos a programa de treinamento com padronização de medidas e estudo-piloto. O banco de dados foi digitado em duplicidade para posterior comparação. Realizou-se o controle de qualidade por meio de instrumento resumido composto de 10 questões, aplicado em 10% da amostra, por contato telefônico ou visita domiciliar.

Foram consideradas como variáveis explicativas: idade (20–29, 30–39, 40–49, 50–59, 60 anos ou mais), cor da pele (branca e não branca), situação conjugal (como ou sem parceiro), escolaridade (4 anos ou menos de estudo, 5 a 9 anos de estudo, 10 a 12 anos de estudo, 13 anos de estudo ou mais), classe econômica (A ou B, C, D ou E), situação laboral (trabalhando, aposentada/afastada/recebendo benefício, dona de casa, desempregada), ser ou não chefe de família, tabagismo (não fumante, ex-fumante ou fumante), etilismo pesado, definido pela ingestão de mais de 30 gramas de álcool/dia (não ou sim), atividade física de lazer, segundo critério do International Physical Activity Questionnaire^23^(IPAQ) (fisicamente ativa ou inativa), gestações prévias (nenhuma, uma e duas ou mais), presença de doenças crônicas, referindo-se a diabetes ou hipertensão arterial (nenhuma doença, alguma das doenças, ou as duas doenças), percepção de saúde (positiva ou negativa) e número de consultas por ano (1 consulta, 2–5, 6–13, 14 ou mais consultas).

Para o presente estudo, foi construído um banco de dados resumido, composto somente pelas variáveis de exposição e pelo instrumento SRQ-20. Após a construção do banco referido, identificaram-se *clusters* a partir dos padrões de respostas do instrumento SRQ-20, por meio de análise de classe latente com o *software* Latent GOLD 5.1. Essa técnica apresenta vantagens em relação às técnicas de clusterização convencionais^24^. Os critérios para definir o número de *clusters* foram o bayesiano (BIC), a porcentagem de erro de classificação e a análise de resíduos^25^.

Optou-se por quatro *clusters* devido ao menor valor de BIC, assim como por um perfil satisfatório entre a porcentagem de erros de classificação e o perfil de resíduos. Além dos critérios mencionados, também se considerou o número proporcional de mulheres em cada *cluster*. A denominação dada aos *clusters* se refere a pontuação média do SRQ-20, assim como aos padrões de resposta das variáveis do instrumento.

Também de modo exploratório, realizou-se uma análise fatorial do instrumento, utilizando matriz tetracórica e valores mínimos de 0,3 e 0,4 para cargas fatoriais e comunalidades, respectivamente. O software utilizado foi o FACTOR 10.9.02. Essa análise fatorial identificou dois fatores (Tabela 1). O primeiro fator, denominado “depressivo”, foi composto pelas variáveis 6 (“Você se sente nervosa, tensa ou preocupada?”), 9 (“Você tem se sentido triste ultimamente?”), 10 (“Você tem chorado mais do que de costume?”), 11 (“Você consegue sentir algum prazer nas atividades diárias?”), 14 (“Você se sente útil na sua vida?”), 15 (“Você tem perdido o interesse pela vida?”), 16 (“Você se sente uma pessoa sem valor?”), 17 (“Você alguma vez pensou em acabar com a sua vida?”), 18 (“Você se sente cansada o tempo todo?”), 20 (“Você se cansa com facilidade?”). Já o segundo fator, denominado “digestivo”, foi composto pelas variáveis 7 (“Você tem má digestão?”) e 19 (“Você sente alguma coisa desagradável no estômago?”).

**Tabela 1:** Análise fatorial utilizando matriz tetracórica.

Variáveis	Fator 1 (Depressivo)	Fator 2 (Digestivo)	Comunalidade
6) Você (a sra.) se sente nervosa, tensa ou preocupada?	0,468	0,267	0,416
7) Você (a sra.) tem má digestão?	-0.108	0,888	0,702
9) Você (a sra.) tem se sentido triste ultimamente?	0,730	0,105	0,622
10) Você (a sra.) tem chorado mais do que de costume?	0.665	0,018	0,455
11) Você (a sra.) consegue sentir algum prazer nas atividades diárias?	-0,848	0,157	0,610
14) Você (a sra.) se sente útil na sua vida?	-0,724	0,083	0,470
15) Você (a sra.) tem perdido o interesse pela vida?	0,936	-0,061	0,822
16) Você (a sra.) se sente uma pessoa sem valor?	0,854	-0,061	0,680
17) Você (a sra.) alguma vez pensou em acabar com sua vida?	0,741	-0,106	0,481
18) Você (a sra.) se sente cansada o tempo todo?	0,674	0,191	0,620
19) Você (a sra.) sente alguma coisa desagradável no estômago?	-0.070	0,971	0,879
20) Você (a sra.) se cansa com facilidade?	0,578	0,284	0,580

O *cluster* 1, ou “baixo”, apresentou um SRQ médio de 1,94 (IC95%: 1,79–2,08), enquanto no *cluster* 4, ou “alto”, a média foi de 13,60 (IC95%: 13,25–13,93). Os *clusters* intermediários (“médio-depressivo” ou 2, e “médio-digestivo” ou 3) apresentaram médias próximas de SRQ: 6,08 (IC95%: 5,87–6,29) e 7,63 (7,36–7,89), respectivamente, porém com um perfil mais positivo para as variáveis representativas do fator depressivo no primeiro grupo e de sintomas digestivos no segundo grupo (Tabela 2). Essas variáveis denominadas “fator depressivo” e “fator digestivo” foram construídas a partir da análise fatorial do instrumento e tiveram valor total máximo de 10 pontos e 2 pontos, respectivamente (Tabelas 1 e 2). Após a identificação dos *clusters*, esse banco foi migrado para os *softwares* SPSS e Stata 8.0, onde foram realizadas análises descritivas da amostra, além de análises brutas e ajustadas.

**Tabela 2:** Comparação entre os SRQ médio e os fatores digestivo e depressivo, segundo a amostra total e os diferentes grupos considerados (n = 1.128).

	Amostra Total (n = 1.128)	SRQ+ (Corte 6/7) (n = 450)	Cluster Baixo (n = 452)	Cluster Médio-Depressivo (n = 240)	Cluster Médio-Digestivo (n = 236)	Cluster Alto (n = 200)
SRQ média (IC95%)	6,07 (5,81–6,34)	10,76 (10,47–11,05)	1,94 (1,79–2,08)	6,08 (5,87–6,29)	7,63 (7,36–7,89)	13,60 (13,25–13,93)
Fator digestivo^a^ (IC95%)	0,64 (0,60–0,69)	1,13 (1,05–1,21)	0,22 (0,17–0,27)	0,03 (0,01–0,04)	1,64 (1,57–1,70)	1,18 (1,06–1,30)
Fator depressivo^b^ (IC95%)	2,86 (2,71–3,01)	5,28 (5,08–5,49)	0,69 (0,62–0,76)	3,30 (3,14–3,46)	2,82 (2,66–2,98)	7,30 (7,10–7,49)

^a^ Fator digestivo: variável com valores entre 0–2, segundo achados fatoriais na amostra.

^b^ Fator depressivo: variável com valores entre 0–10, segundo achados fatoriais na amostra.

Em relação às variáveis de resposta (grupos-resposta), foram contemplados 5 grupos. O primeiro foi o grupo positivo para TMC, considerando o ponto de corte 6/7^10^. Utilizou-se esse grupo a modo de referência, como uma forma mais clássica de investigar fatores associados em relação ao assunto. Os outros quatro grupos-resposta foram os quatro *clusters* descritos. Avaliou-se a associação entre as variáveis explicativas e as variáveis de resposta (grupos-resposta) pelas razões de prevalência (RP) e pelos intervalos de confiança (IC95%). As análises brutas e ajustadas foram realizadas por meio da regressão de Poisson, com controle para efeito de delineamento. Nas análises ajustadas, incluíram-se inicialmente todas as variáveis, retirando sequencialmente das análises aquelas que não apresentaram valor-p mínimo de 0,05. Por fim, permaneceram somente as variáveis significativas em cada um dos cinco grupos-resposta considerados.

## RESULTADOS

Ao considerar os diferentes *clusters*, 40,1% das mulheres pertenceram ao *cluster* denominado “baixo”, 21,3% ao *cluster* “médio-depressivo”, 20,9% ao “médio-digestivo”, e 17,7% ao *cluster* “alto”. Como mostra a Tabela 3, 39,9% das mulheres apresentaram critério de TMC positivo, segundo o ponto de corte 6/7 no instrumento SRQ-20 (Tabela 3).

**Tabela 3:** Análise descritiva das variáveis socioeconômicas, demográficas, comportamentais e de saúde e doença na amostra de mulheres do município de São Leopoldo, Sul do Brasil (n = 1.128).

Grupos	n	% SRQ+ (Corte 6/7)	% Cluster Baixo	% Cluster Médio-Depressivo	% Cluster Médio-Digestivo	% Cluster Alto
Variáveis	1.128	39,9	40,1	21,3	20,9	17,7
Idade (anos)						
20–29	216	18,2	20,1	**18,3**	19,9	17,0
30–39	244	24,2	16,8	**30,8**	24,6	18,0
40–49	276	23,8	24,3	**24,6**	22,9	26,5
50–59	228	19,3	23,0	**14,2**	18,2	23,5
60 ou mais	164	14,4	15,7	**12,1**	14,4	15,0
Cor da pele						
Branca	840	72,7	76,3	72,9	74,2	72,5
Não branca	288	27,3	23,7	27,1	25,8	27,5
Situação conjugal						
Com parceiro	720	62,9	62,4	67,9	65,3	60,5
Sem parceiro	408	37,1	37,6	32,1	34,7	39,5
Escolaridade (anos)						
13 ou mais	114	**4,8**	**14,6**	8,0	11,0	**2,1**
10–12	391	**30,6**	**38,4**	35,9	35,2	**27,1**
5–9	417	**40,4**	**34,8**	40,5	32,6	**46,4**
4 ou menos	188	24,3	12,1	15,6	21,2	24,5
Classe econômica						
A ou B	390	**25,7**	**40,6**	35,7	35,2	**20,0**
C	596	**58,0**	**49,8**	53,4	54,2	**59,0**
D ou E	136	**16,3**	**9,6**	10,9	10,6	**21,0**
Situação laboral						
Trabalhando	637	**50,3**	**61,9**	56,5	58,5	**42,5**
Aposent./afast.^a^	186	**16,7**	**17,7**	13,8	14,4	**19,5**
Dona de casa	182	**18,7**	**13,5**	17,6	14,0	**23,0**
Desempregada	121	**14,3**	**6,9**	12,1	13,1	**15,0**
Chefe de família						
Não	544	**44,0**	50,9	50,0	47,5	**41,0**
Sim	584	**56,0**	49,1	50,0	52,5	**59,0**
Tabagismo						
Não	661	**54,0**	**62,4**	61,3	58,5	**47,0**
Ex-tabagista	259	**21,8**	**23,9**	21,3	26,3	**19,0**
Sim	208	**24,2**	**13,7**	17,5	15,3	**34,0**
Etilismo pesado						
Não	1087	95,8	97,3	95,8	98,3	96,0
Sim	34	4,2	2,7	4,2	1,7	4,0
Atividade física						
Sim	162	**8,4**	**20,8**	12,1	12,7	**4,5**
Não	966	**91,6**	**79,2**	87,9	87,3	**95,5**
Gestações						
Nenhuma	172	**11,3**	**18,8**	15,4	15,3	**7,0**
1	254	**19,8**	**23,7**	25,4	22,0	**17,0**
2 ou mais	702	**68,9**	**57,5**	59,2	62,7	**76,0**
Doenças crônicas						
Nenhuma	785	**62,7**	**75,5**	75,3	69,8	**51,8**
Uma	269	**28,8**	**20,5**	18,4	24,7	**38,0**
Duas	66	**8,5**	**4,0**	6,3	5,5	**10,2**
Percepção saúde						
Positiva	748	**45,6**	**83,4**	66,3	62,7	**32,0**
Negativa	380	**54,4**	**16,6**	33,8	37,3	**68,0**
Consultas/ano						
Nenhuma	174	**16,0**	**14,6**	18,8	**10,2**	19,6
1	179	**11,4**	**20,6**	15,0	**11,9**	11,1
2–5	277	**19,6**	**27,9**	23,3	**27,5**	15,0
6–13	172	**17,1**	**14,4**	14,6	**15,7**	17,6
14 ou mais	325	**35,9**	**22,6**	28,3	**34,7**	36,7

^a^ Aposent./afast.: categoria que compreendeu mulheres aposentadas, afastadas, recebendo benefício ou licença-maternidade. Os valores em negrito são referentes a variáveis significativas (p < 0,05) em cada grupo considerado.

Em relação às variáveis socioeconômicas e demográficas, houve predomínio de idades intermediárias, entre 30 e 49 anos, quando considerado o grupo do ponto de corte. Já em relação aos *clusters*, as idades mais elevadas tenderam a ser mais prevalentes naquelas mulheres com SRQ-20 mais elevado. A cor da pele branca predominou em todos os grupos, assim como a situação conjugal de ter parceiro. Ao se analisar a escolaridade, o grupo com maior estudo (13 anos ou mais) foi o menos prevalente no grupo com SRQ+ em relação ao ponto de corte. Nos *clusters*, a escolaridade variou inversamente com a maior pontuação do SRQ-20. As classes econômicas menos privilegiadas (D ou E) foram mais prevalentes no *cluster* “alto”, sendo a classe C a mais prevalente em todos os *clusters*, assim como quando considerado o ponto de corte. Em relação à situação laboral, a condição de estar trabalhando prevaleceu em todos os grupos, e a distribuição de mulheres com a condição de ser chefe de família foi similar, com ligeiro aumento da prevalência nos grupos do ponto de corte e no *cluster* “alto” (Tabela 3).

Ao se considerar as variáveis comportamentais, as mulheres não tabagistas e fisicamente inativas predominaram em todos os grupos. O etilismo pesado (> 30g de álcool/dia) foi muito pouco prevalente em todos os grupos (Tabela 3).

A maioria das mulheres relatou não apresentar nenhuma doença crônica (diabetes ou hipertensão arterial). A maior parte também relatou duas ou mais gestações. A percepção de saúde positiva variou inversamente com os *clusters* em relação à maior pontuação no SRQ-20: a maior frequência (83,4%) foi encontrada no *cluster* “baixo”, e a menor (32%) no *cluster* “alto”. O indicador do número de consultas/ano variou conforme o grupo, distribuindo-se de maneira mais irregular (Tabela 3).

Nas análises brutas e ajustadas, no *cluster* de maior proteção para os TMC, que foi denominado “baixo”, encontrou-se relação entre idade, escolaridade, situação laboral, atividade física, percepção de saúde e número de consultas/ano (Tabela 4). A classe econômica, o tabagismo, a atividade física, a presença de doenças crônicas e a percepção de saúde estiveram associados ao *cluster* “alto” (Tabela 4). Nos *clusters* intermediários, somente dois fatores foram identificados: a idade no *cluster* “médio-depressivo”, e o número de consultas/ano no *cluster* “médio-digestivo” (Tabela 5).

**Tabela 4:** Análises brutas e ajustadas dos grupos com SRQ+ segundo o ponto de corte 6/7, *cluster* “baixo” e *cluster* “alto”.

Grupos	SRQ + (Corte 6/7)		Cluster 1 (Baixo)		Cluster 4 (Alto)	
		
	RP Bruta	RP Ajustada	RP Bruta	RP Ajustada	RP Bruta	RP Ajustada
Variáveis						
Idade (anos)						
20–29	1	1	1	1		
30–39	1,18 (0,92–1,51)	1,20 (0,94–1,53)	0,74 (0,57–0,96)	0,73 (0,56–0,95)		
40–49	1,02 (0,79–1,32)	0,90 (0,71–1,16)	0,95 (0,76–1,17)	0,98 (0,79–1,21)		
50–59	1,01 (0,74–1,37)	0,79 (0,60–1,02)	1,08 (0,85–1,37)	1,24 (1,00–1,53)		
60 ou mais	1,04 (0,79–1,39)	0,73 (0,54–0,99)	1,03 (0,79–1,33)	1,23 (0,93–1,61)		
Escolaridade (anos)						
13 ou mais	1	1	1	1		
10–12	1,87 (1,23–2,86)	1,59 (1,07–2,37)	0,77 (0,64–0,92)	0,90 (0,75–1,08)		
5–9	2,32 (1,48–3,62)	1,71 (1,12–2,60)	0,65 (0,54–0,78)	0,84 (0,70–1,00)		
4 ou menos	3,09 (1,92–4,98)	2,20 (1,44–3,35)	0,50 (0,35–0,73)	0,68 (0,48–0,95)		
Classe econômica						
A ou B						
C					1,93 (1,31–2,85)	1,16 (0,80–1,69)
D ou E					3,01 (2,05–4,43)	1,76 (1,19–2,58)
Situação laboral						
Trabalhando			1	1		
Aposent./afast.^a^			0,98 (0,84–1,15)	1,06 (0,91–1,24)		
Dona de casa			0,77 (0,59–1,00)	0,88 (0,67–1,15)		
Desempregada			0,58 (0,43–0,80)	0,70 (0,53–0,92)		
Tabagismo						
Não	1	1			1	1
Ex-tabagista	1,03 (0,87–1,22)	0,97 (0,82–1,14)			1,03 (0,77–1,38)	0,92 (0,69–1,22)
Sim	1,43 (1,24–1,65)	1,13 (0,99–1,31)			2,30 (1,79–2,95)	1,67 (1,30–2,16)
Etilismo pesado						
Não	1	1				
Sim	1,42 (1,05–1,90)	1,41 (1,11–1,79)				
Atividade física						
Ativo	1	1	1	1	1	1
Inativo	1,82 (1,33–2,49)	1,35 (1,00–1,82)	0,64 (0,54–0,76)	0,78 (0,67–0,92)	3,56 (1,87–6,77)	2,19 (1,19–4,02)
Doenças crônicas						
Nenhuma					1	1
Uma					2,15 (1,61–2,87)	1,49 (1,12–1,98)
Duas					2,33 (1,44–3,78)	1,36 (0,84–2,21)
Percepção saúde						
Positiva	1	1	1	1	1	1
Negativa	2,35 (2,06–2,68)	2,09 (1,86–2,34)	0,39 (0,32–0,49)	0,44 (0,35–0,55)	4,18 (3,15–5,55)	3,14 (2,31–4,27)
Consultas/ano						
Nenhuma	1	1	1	1		
1	0,69 (0,52–0,91)	0,74 (0,55–1,01)	1,37 (1,08–1,74)	1,29 (1,02–1,64)		
2–5	0,77 (0,61–0,96)	0,87 (0,71–1,07)	1,20 (0,96–1,50)	1,08 (0,86–1,36)		
6–13	1,08 (0,87–1,34)	1,21 (0,98–1,50)	1,00 (0,75–1,33)	0,93 (0,70–1,22)		
14 ou mais	1,20 (0,96–1,50)	1,19 (0,95–1,50)	0,83 (0,63–1,09)	0,83 (0,63–1,09)		

RP: razão de prevalência.

^a^ Aposent./afast.: categoria que compreendeu mulheres aposentadas, afastadas, recebendo benefício ou licença-maternidade.

**Tabela 5:** Fatores associados em relação aos *clusters* intermediários.

Grupos	Cluster 2 (Médio-Depressivo)		Cluster 3 (Médio-Digestivo)	
	
	RP Bruta	RP Ajustada	RP Bruta	RP Ajustada
Variáveis				
Idade^a^ (anos)				
20–29	1	1		
30–39	1,49 (1,06–2,09)	1,49 (1,06–2,09)		
40–49	1,05 (0,73–1,51)	1,05 (0,73–1,51)		
50–59	0,73 (0,48–1,12)	0,73 (0,48–1,12)		
60 ou mais	0,87 (0,57–1,33)	0,87 (0,57–1,33)		
Consultas/ano^b^				
Nenhuma			1	1
1			1,13 (0,69–1,85)	1,13 (0,69–1,85)
2–5			1,70 (1,08–2,67)	1,70 (1,08–2,67)
6–13			1,56 (0,96–2,52)	1,56 (0,96–2,52)
14 ou mais			1,83 (1,12–2,98)	1,83 (1,12–2,98)

RP: razão de prevalência.

^a^ Única variável significativa no *cluster* 2.

^b^ Única variável significativa no *cluster* 3.

Quando considerado o grupo do ponto de corte, foi encontrada relação entre idade, escolaridade, tabagismo, etilismo, atividade física, percepção de saúde e número de consultas/ano. Nesse grupo, as menores razões de prevalência foram encontradas nas mulheres mais idosas e, na categoria de idade entre 30 e 39 anos, evidenciou-se uma prevalência 20% maior que a categoria de referência (ter entre 20 e 29 anos). No *cluster* “baixo”, houve tendência consistente de aumento da prevalência de mulheres com o aumento da idade, com razão de prevalência de 1,24 no grupo de mulheres entre 50 e 59 anos (Tabela 4).

A escolaridade apresentou-se como fator associado relevante quando considerado o ponto de corte, com prevalência 2,2 vezes maior no grupo entre 0 e 4 anos de estudo em comparação à categoria de referência de haver estudado 13 anos ou mais. No *cluster* “baixo”, essa variável também foi significativa, ficando constatada a menor razão de prevalência (0,68) entre as mulheres com menor estudo, o que demonstra efeito protetor da variável em relação aos TMC. Já a classe econômica apresentou associação somente no *cluster* “alto”, com razão de prevalência de 1,76 nas classes mais baixas (D ou E). Outra variável socioeconômica relacionada, a situação laboral, evidenciou tendência linear inversa no sentido de trabalho-desemprego no *cluster* “baixo”, com menor razão de prevalência (0,70) e maior significância entre as desempregadas (Tabela 4).

Na variável comportamental que considerou o tabagismo, as razões de prevalência foram significativas na categoria dos tabagistas, tanto no grupo do ponto de corte (RP = 1,13) quanto no *cluster* “alto” (RP = 1,67). Já o etilismo pesado (consumo > 30 gramas de álcool/dia) somente esteve associado ao grupo do ponto de corte, com uma razão de prevalência de 1,41.

Em relação à atividade física, houve associação entre ser inativo fisicamente nos grupos do ponto de corte (RP = 1,35), *cluster* “baixo” (RP = 0,78) e *cluster* “alto” (RP = 2,19), com todos os intervalos de confiança consistentes (Tabela 4). Da mesma forma e com significância, evidenciou-se também associação entre a variável percepção de saúde nos três grupos. A menor razão de prevalência de uma percepção negativa foi encontrada no *cluster* “baixo” (RP = 0,44), e a maior no *cluster* “alto” (RP = 3,14), com valor intermediário no grupo do ponto de corte (RP = 2,09) (Tabela 4).

A presença de doenças crônicas apresentou significância somente no *cluster* “alto”, com prevalência ligeiramente maior na categoria “apresentar uma doença crônica” (RP = 1,49) em comparação a “apresentar duas” (RP = 1,36) (Tabela 4).

No que se refere às consultas médicas, no grupo do ponto de corte constatou-se tendência de prevalências menores, em relação à referência (nenhuma consulta), nas categorias entre 1 e 5 consultas; e prevalências crescentes em relação a um maior número de consultas (6 ou mais consultas). No *cluster* “baixo”, houve significância nas categorias de uma consulta, e de 14 ou mais consultas, com razões de prevalência de 1,29 e 0,83, respectivamente (Tabela 4). No *cluster* “médio-digestivo”, houve tendência de razões de prevalência progressivas no mesmo sentido das categorias com maior número de consultas. O maior valor encontrado (RP = 1,83) foi aquele correspondente à categoria com mais consultas: 14 ou mais (Tabela 5).

Também foram investigadas as médias do SRQ-20 em cada um dos grupos analisados, assim como dos fatores digestivo (variáveis 7 e 19) e depressivo (variáveis 6, 9, 10, 11, 14, 15, 16, 17, 18 e 20), definidos segundo análise fatorial da amostra (Tabelas 1 e 2). Em relação ao fator digestivo, observou-se que o *cluster* “médio-digestivo” foi o que apresentou a maior pontuação nesse fator, maior inclusive que o *cluster* “alto”. Já o *cluster* “médio-depressivo” foi o que apresentou a menor pontuação nesse fator, menor até que o *cluster* “baixo” (Tabela 2).

## DISCUSSÃO

A literatura aponta associação significativa entre TMC e o sexo feminino. Os transtornos de humor e a ansiedade são mais prevalentes em mulheres em comparação com os homens, que por sua vez têm mais chances de apresentar transtornos por uso de substâncias^4^. Estudos que pretendem identificar fatores associados aos TMC com instrumentos de rastreamento de saúde mental e seus padrões de resposta, partindo de bases populacionais específicas de mulheres, agregam dados a esse contexto. Quando considerada a visão clássica da análise dos fatores associados aos TMC a partir de um ponto de corte, deve-se ter presente que, dentro dos grupos sem presença de doença (SRQ-) ou com presença de doença (SRQ+) – no caso do presente estudo representada pela morbidade psiquiátrica –, podem existir diferentes subgrupos ou *clusters*, com características particulares e fatores associados específicos.

A utilização do tradicional ponto de corte pode fazer que fatores associados sejam atribuídos ou identificados em grupos que, mesmo sendo muito heterogêneos, são “homogeneizados” por essa metodologia. A avaliação por subgrupos ou *clusters* baseados nos padrões de respostas do SRQ-20 possibilitou vislumbrar em qual grupo cada fator associado apresentou relevância. Essa nova visão, portanto, pode trazer mais especificidade aos estudos sobre TMC e fatores associados, questionando o tradicional ponto de corte.

Ao considerar a idade como fator associado aos TMC, a literatura tem demonstrado resultados contraditórios^2,3,12,13,21^. A maioria dos estudos revisados observou aumento consistente da prevalência de TMC com a idade, o que não foi constatado no presente estudo. Os resultados aqui encontrados provavelmente refletem as demandas crescentes e o aumento do estresse entre mulheres mais jovens^13^. Devido à análise utilizada, que contemplou *clusters*, constatou-se efeito protetor da idade em relação aos TMC, com tendência de aumento da presença de mulheres com idades mais elevadas no grupo de menor pontuação do SRQ-20 (*cluster* “baixo”). Já em *clusters* com maior probabilidade de apresentar TMC, a tendência se mostrou inversa. Assim, houve redução das prevalências de mulheres com idades mais avançadas a medida que as pontuações no SRQ-20 progrediram (*cluster* “médio-depressivo” e grupo do ponto de corte).

Estudos têm mostrado que desvantagens sociais como baixo nível educacional, renda, classe econômica e desemprego permanecem sendo os fatores de risco mais consistentes para os TMC^2,3,12,14^. Apesar da classe econômica e da escolaridade serem variáveis relacionadas, neste estudo foi constatada influência diferente dessas duas variáveis quando avaliados os *clusters*. Assim, a maior escolaridade apresentou efeito protetor para os TMC, evidenciado no *cluster* “baixo”, assim como o contexto de histórico laboral. A literatura descreve que, entre as mulheres, haveria um efeito protetor do ato de trabalhar sobre os TMC, em comparação com a condição de ser dona de casa ou estar desempregada^3,21^. A classe econômica foi a única variável socioeconômica identificada no grupo com maior probabilidade de morbidade psiquiátrica (*cluster* “alto”), revelando tendência de maiores prevalências entre as classes econômicas mais baixas, com razão de prevalência significativa de 1,76 nas classes D ou E.

Já no grupo do ponto de corte, somente a escolaridade teve importância como variável socioeconômica, com evidência de aumento das prevalências de maneira consistente à medida que a escolaridade diminui. Existiria, portanto, maior sofrimento mental entre mulheres com piores condições econômicas ou desfavorecidas socialmente. Somadas essas informações, pode-se supor que menores taxas de desemprego, aumento da escolaridade e ascensão às classes econômicas mais favorecidas melhorariam a saúde mental. Cada uma dessas variáveis apresenta uma influência diferente em cada grupo considerado.

Em relação ao tabagismo, a análise apresentou significância na categoria das mulheres atualmente tabagistas no grupo do ponto de corte, corroborando outros estudos realizados no Sul do Brasil^13,20^. No *cluster* “alto”, também foi encontrada essa associação, porém com maior magnitude. Assim, infere-se que a influência do tabagismo se deu sobretudo entre mulheres com o SRQ mais elevado, e não nos valores intermediários, como poderia sugerir a metodologia do ponto de corte.

Estudos também identificaram o etilismo e sua relação com os TMC^13,20,26^. Com o critério do ponto de corte, houve associação significativa entre os TMC e a variável comportamental do etilismo pesado (> 30g de álcool/dia). Na análise segundo os *clusters*, essa relação não foi evidenciada, demonstrando que a análise baseada no tradicional ponto de corte encontrou resultado diferente devido ao fato de submeter mulheres com diferentes valores de SRQ+ (que poderiam variar desde 7 até 20) a uma mesma análise.

As variáveis de atividade física e de percepção de saúde foram significativas tanto no grupo do ponto de corte quanto nos *clusters* extremos (“baixo” e “alto”). Conforme descrito na literatura, encontrou-se efeito benéfico da atividade física em relação ao bem-estar físico e psicossocial, o que se reflete nas razões de prevalência no *cluster* “baixo”. O incremento muscular e a redução da porcentagem de gordura, a otimização das condições cardiorrespiratórias, a redução da ansiedade e da depressão – que impactam no humor e na autoestima – seriam alguns dos efeitos protetores contra os TMC e diversas doenças crônicas^13^. Corroborando outros estudos, foi encontrada maior prevalência de mulheres fisicamente inativas nos grupos do ponto de corte e no *cluster* “alto”, que apresentaram maior morbidade psiquiátrica^13,26^. A autopercepção de saúde considerada negativa apresentou maiores razões de prevalência nos grupos com maior pontuação no SRQ-20 (ponto de corte e *cluster* “alto”), indo ao encontro dos achados da literatura^2,13,21^.

Em relação a presença de doenças crônicas, pesquisa internacional realizada pela Organização Mundial da Saúde (OMS) em 17 países revelou associação entre os TMC e diversas patologias crônicas como diabetes, asma, hipertensão, artrite, úlcera e doenças cardíacas^27^. Apesar da literatura apontar associação entre os TMC e condições crônicas com o tradicional ponto de corte, o presente estudo observou associação com essas condições apenas no grupo com SRQ mais elevado (“alto”). Esse achado sugere que medidas que otimizem o manejo de doenças crônicas podem ter impacto entre mulheres com maior morbidade psiquiátrica. No *cluster* “alto”, ficou claramente caracterizada a concomitância de fatores comportamentais (tabagismo e inatividade física) com variáveis de saúde e doença (autopercepção de saúde negativa e presença de doenças crônicas).

O *cluster* com maior pontuação no fator digestivo (“médio-digestivo”) apresentou relação progressiva com o número de consultas. Segundo Bekhuis et al.^28^, existem diferentes combinações entre sintomas depressivos, ansiosos e somáticos, o que impacta na demanda por serviços de saúde. Nessa amostra, os sintomas digestivos motivaram um maior número de consultas médicas do que a própria pontuação do SRQ-20 ou sintomas depressivos. Assim, deveria haver um olhar especial para esses sintomas digestivos, atentando para interpretações complexas decorrentes dos sintomas expressados, de modo a compreender melhor essa demanda.

Em resumo, considerando os fatores associados encontrados, o *cluster* “baixo”é formado majoritariamente por mulheres de idade mais elevada, com maior escolaridade e histórico laboral, fisicamente ativas, com autopercepção de saúde positiva e tendência de se consultar poucas vezes com o médico. O *cluster* “alto” é composto por mulheres pertencentes a classes sociais mais baixas, tabagistas, inativas fisicamente, com presença de doenças crônicas e autopercepção de saúde negativa. Mulheres com tendência de procurar mais por consultas médicas, valores de SRQ-20 intermediário e maior expressão de sintomas digestivos em geral pertencem ao *cluster* médio-digestivo. Já o *cluster* médio-depressivo, também com valores intermediários no SRQ-20, é caracterizado por mulheres jovens, com relato de sintomas mais condizentes com esfera depressiva. O grupo do ponto de corte é formado por mulheres mais jovens, com menor escolaridade, etilistas pesadas e tabagistas, inativas fisicamente, com autopercepção de saúde ruim e tendência maior de procura por consultas médicas.

Como limitações do presente estudo, pode-se mencionar a impossibilidade de se estender seus achados à população em geral, haja vista que a amostra esteve restrita a mulheres. Há também possibilidade de causalidade reversa, visto que se trata de estudo de delineamento transversal. Apesar disso, estudos desse tipo são ferramentas importantes para identificar e descrever grupos de risco, contribuindo para o planejamento de ações em saúde^29,30^.

## CONCLUSÃO

Este estudo, que utilizou a análise de *cluster* por classe latente, apresenta uma perspectiva nova e diferenciada em relação aos TMC e fatores associados. A caracterização de subgrupos, definidos segundo o perfil de resposta do instrumento SRQ-20, permitiu encontrar fatores associados particulares, dando mais especificidade aos resultados. Nessa perspectiva e considerando a relevância do assunto, são necessários mais estudos com essa metodologia.
